# A Comparison of Functional Features in Chinese and US Mobile Apps for Diabetes Self-Management: A Systematic Search in App Stores and Content Analysis

**DOI:** 10.2196/13971

**Published:** 2019-08-28

**Authors:** Yuan Wu, Yiling Zhou, Xuan Wang, Qi Zhang, Xun Yao, Xiaodan Li, Jianshu Li, Haoming Tian, Sheyu Li

**Affiliations:** 1 Department of Endocrinology and Metabolism West China Hospital Sichuan University Chengdu China; 2 Xi An Jiao Tong University The First Affiliated Hospital of Xi An Jiao Tong University Xi An China; 3 Department of Population Health & Genomics Ninewells Hospital and Medical School University of Dundee Dundee United Kingdom; 4 Science for Life Laboratory, Department of Medical Cell Biology Uppsala University Uppsala Sweden; 5 State Key Laboratory of Ophthalmology, Zhongshan Ophthalmic Center Sun Yat-sen University Guangzhou China; 6 Department of Academic Affairs West China School of Medicine Sichuan University Chengdu China; 7 Department of Gastroenterology West China Hospital Sichuan University Chengdu China; 8 Medicines Monitoring Unit Ninewells Hospital and Medical School University of Dundee Dundee United Kingdom; 9 Department of Biomedical Polymer and Artificial Organs College of Polymer Science and Engineering Sichuan University Chengdu China

**Keywords:** diabetes mellitus, self-management, mobile apps, risk assessment, prevalence, China, United States

## Abstract

**Background:**

Mobile health interventions are widely used for self-management of diabetes, which is one of the most burdensome noncommunicable chronic diseases worldwide. However, little is known about the distribution of characteristics and functions of in-store mobile apps for diabetes.

**Objective:**

This study aimed to investigate the distribution of characteristics and functions of the in-store mobile apps for self-management of diabetes in the United States and China using a predefined functional taxonomy, which was developed and published in our previous study.

**Methods:**

We identified apps by searching diabetes in English or Chinese in the Apple iTunes Store and Android Markets (both in the United States and China) and included apps for diabetes self-management. We examined the validity and reliability of the predefined functional taxonomy with 3 dimensions: clinical module, functional module, and potential risk. We then classified all functions in the included apps according to the predefined taxonomy and compared the differences in the features of these apps between the United States and China.

**Results:**

We included 171 mobile diabetes apps, with 133 from the United States and 38 from China. Apps from both countries faced the challenges of evidence-based information, proper risk assessment, and declaration, especially Chinese apps. More Chinese apps provide app-based communication functions (general communication: Chinese vs US apps, 39%, 15/38 vs 18.0%, 24/133; *P*=.006 and patient-clinician communication: Chinese vs US apps, 68%, 26/38 vs 6.0%, 8/133; *P*<.001), whereas more US apps provide the decision-making module (Chinese vs US apps, 0%, 0/38 vs 23.3%, 31/133; *P*=.001), which is a high-risk module. Both complication prevention (Chinese vs US apps, 8%, 3/38 vs 3.8%, 5/133; *P*=.50) and psychological care (Chinese vs US apps, 0%, 0/38 vs 0.8%, 1/133; *P*>.99) are neglected by the 2 countries.

**Conclusions:**

The distribution of characteristics and functions of in-store mobile apps for diabetes self-management in the United States was different from China. The design of in-store diabetes apps needs to be monitored closely.

## Introduction

### Background

Diabetes mellitus is one of the most burdensome noncommunicable chronic diseases in China and the western world [1-3]. It represented 8.4% of all-cause deaths worldwide among adults aged 20 to 79 years in 2013 [4] and incurred a cost of US $1.31 trillion in 2015 [5].

Diabetes requires long-term care that is highly individualized to suit the needs of each patient [6]. The care includes lifestyle modification (eg, diet, physical activity, and weight management), glucose monitoring, prevention of complications, and multiple medication regimens [7,8]. Barriers to the effectiveness of diabetes care include insufficient knowledge and training that patients received [9], requiring permanent behavior change [10], financial burden [11], and complication-specific treatments [12]. Patient education and support by a multidisciplinary team of professionals (eg, physicians, nurses, dietitians, and psychotherapists) may help improve the quality of care [13], although they could be costly and unavailable in some developing countries [14].

Mobile apps developed for diabetes self-management are new and become more and more popular [15-19]. Although randomized trials and systematic reviews suggest that mobile apps, in general, are effective in improving the glucose control for patients with type 2 diabetes [20], the apps available in app stores are highly varied in function, design, and quality [21] and have not been necessarily assessed rigorously by effective randomized control trials. Our previous study [8] developed a taxonomy with 3 dimensions (ie, clinical module, functional module, and potential risk) to provide a functional classification and risk assessment for mobile apps for diabetes self-management and suggested that different functions and combinations may contribute differently to the effectiveness of diabetes management. The clinical module of this taxonomy consists of monitoring, medication management, lifestyle modification, complication prevention, and psychosocial care; the functional module includes logs, structured display, general education, personalized feedback, and communication; potential risk includes 3 levels: high, medium, and low risk. The taxonomy serves as a novel and reliable tool to classify and evaluate the content and functions of diabetes self-management apps in the market.

### Objectives

This study aimed to describe and compare the characteristics and functions of mobile apps for diabetes self-management in 2 of the largest app markets, China and the United States, to provide suggestions for future development and usage of mobile apps for diabetes self-management using our predefined taxonomy [8].

## Methods

### Data Source

In an electronic search, conducted on December 5, 2016, using *diabetes* as the keyword in both English and Chinese languages, we identified apps in English or Chinese from Apple iTunes store (China and the United States), Google Android Play (the United States), Tencent Android Market (Tencent Holdings Limited), Baidu Android Market (Baidu, Inc), and 360 Android Market (Qihoo 360 Technology Co Ltd).

### Selection Criteria

We included mobile apps for diabetes self-management, which was defined as supporting the interactive self-monitoring of blood glucose. The first 500 mobile apps in the initial search list were included in this study. This is because users tend to choose the top mobile apps that are sorted based on customer reviews and download count. Mobile apps outside the range are less likely to be selected and downloaded by users.

The exclusion criteria were (1) duplicated apps (apps with same name and same producer are defined as duplication regardless of different versions), (2) apps without any meaningful introduction or instruction in the app store, (3) apps designed only for the health professionals, (4) apps without Chinese or English interfaces, and (5) apps with no update since January 1, 2014.

### App Selection and Data Extraction

In total, 2 investigators (YW and QZ) independently selected the apps for inclusion according to the selection criteria. The investigators extracted the following data from each included study: the app name, developer, specifications (medical, health, and fitness or unavailable), acquisition cost (free, paid, or in-app purchase), downloading fee, the latest update date, target users (type 1 diabetes, type 2 diabetes, gestational diabetes, prediabetes, all types, or unavailable), safety statement (potential risks or use under guidance), supporting evidence (descriptive study, observational study, or randomized controlled trials), and source of information (clinical guidelines). The inconsistency of the study inclusion and data extraction was solved by a discussion between the 2 investigators.

### Validity and Reliability of the Developed Taxonomy

The list of function was summarized according to our previously developed taxonomy, which was described in detail in previous publication [8]. First, the preliminary taxonomy was coded [8]. Then 2 reviewers independently classified functions of all included apps according to the coded preliminary taxonomy and slightly modified the preliminary taxonomy. Furthermore, 2 new functions, namely recording insulin injection and reminder to record the medication, were added to the slot of recording used medication and side effect and to the slot of reminder to take the medication of the taxonomy, respectively (Multimedia Appendix 1). The function of off-target alert and setting targets were moved to the complication prevention slot because it is more than a monitoring function but a strategy for complication prevention. Diabetes process and treatment options were removed from the slot of using medications safely and effectively. This process guaranteed that our predefined taxonomy could be used to classify all current in-store diabetes apps. A panel of 5 reviewers used the predefined taxonomy to classify functions of 10 Chinese apps and 10 US apps, which covered all coded functions to assess the reliability of taxonomy with Krippendorff alpha.

### Assessment of the App Functions

Functional features of included apps were specified by crossing of the functional and clinical module with a risk assessment for each slot [8]. The potential risk of each function was assessed using methods based on the taxonomy, which was developed according to the US Food and Drug Administration (FDA) risk-based framework [8,22]. The risk level of a mobile app was determined by the highest risk level of any of its functions.

### Statistics Analysis

The baseline characteristics were summarized using SPSS (version 21), and the comparison between the Chinese and US apps was evaluated using a chi-square test or Fisher exact test with a significance level of .05 using Open Epi (version 3.01). The frequencies and percentages of the modules were calculated using SPSS (version 21). The functional differences between the Chinese and US apps were examined by a chi-square test with a significance level of .05 by Open Epi (version 3.01).

## Results

### Basic Characteristics

**Figure 1:**
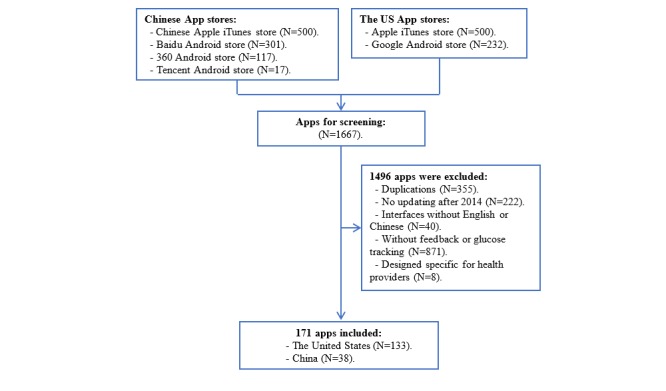
The flowchart of app selection.

After searching in the Apple iTunes store, Google Android store, Baidu Android Store, 360 Android Store, and Tencent Android Store, 1667 apps were found. After screening, 171 apps were finally included in this study, 38 from China and 133 from the United States. The process of app selection is shown in the flowchart in Figure 1.

The characteristics of all included apps were summarized in Table 1. Among Chinese mobile apps, 21% (8/38) were not adequately categorized (*P*<.001), whereas all the 133 US mobile apps were categorized as either *health and fitness* or *medical* mobile apps. Regarding the acquisition costs, there were more free apps available in China than in the United States (92% [35/38] in China vs 75.2% [100/133] in the United States; *P*=.04). US mobile apps had more specific target audiences than Chinese mobile apps (less than 3% [1/38] in China vs 21.1% [28/133] in the United States; *P*=.01). None of the Chinese mobile apps provided a clear safety declaration or supporting evidence, whereas 14.3% (19/133) of US mobile apps had a clear safety declaration, and 2.2% (3/133) of US mobile apps supplied supporting evidence.

**Table 1:** Metadata of in-store mobile apps for diabetes self-management between the United States and China.

Category			China (N=38)	United States (N=133)	*P* value
**Specifications, n (%)**					
	Medical		4 (11)	90 (67.7)	<.001^a^
	Health fitness		26 (68)	43 (32.3)	<.001^a^
	N/A^b^		8 (21)	0	<.001^a^
**Acquisition costs, n (%)**					
	Free		35 (92)	100 (75.2)	.04^a^
	In-app purchase		0	22 (16.5)	.04^a^
	Paid		3 (8)	11 (8.3)	.04^a^
**Statement on target users, n (%)**					
	**With clear statement**				
		T1DM^c^	0	2 (1.5)	.01^a,d^
		T2DM^e^	0	6 (4.5)	.01^a,d^
		GDM^f^	1 (3)	1 (0.8)	.01^a,d^
		All types	0	19 (14.3)	.01^a,d^
	Without clear statement		37 (97)	105 (78.9)	.01^a,d^
**Safety statement, n (%)**					
	With		0	19 (14.3)	.03^g^
	Without		38	114 (85.7)	.03^g^
**Supporting evidence, n (%)**					
	With		0	3 (2.2)	.99^g^
	Without		38	130 (97.8)	.99^g^

^a^Chi-square test.

^b^N/A: not available.

^c^T1DM: type 1 diabetes.

^d^Compare apps with a clear statement on target users between China and the United States.

^e^T2DM: type 2 diabetes.

^f^GDM: gestational diabetes mellitus.

^g^Fisher exact test.

### Validity and Reliability of Taxonomy

The standardized classification code was completed, which supported the validity of the taxonomy. The inconsistency of coding by each coding investigator was presented as the standardized rate of coding error (Multimedia Appendix 2). The Krippendorff alpha is .8229, which shows the reliability of the taxonomy is acceptable.

### Functions and Modules

**Figure 2:**
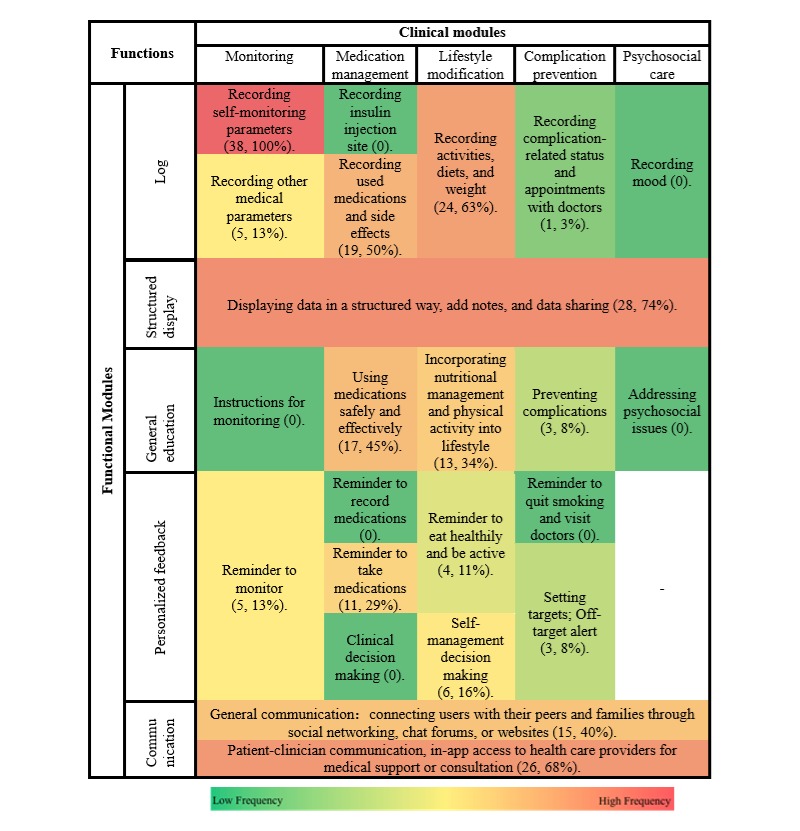
Heatmap of features of 38 Chinese mobile apps for diabetic self-management.

**Figure 3:**
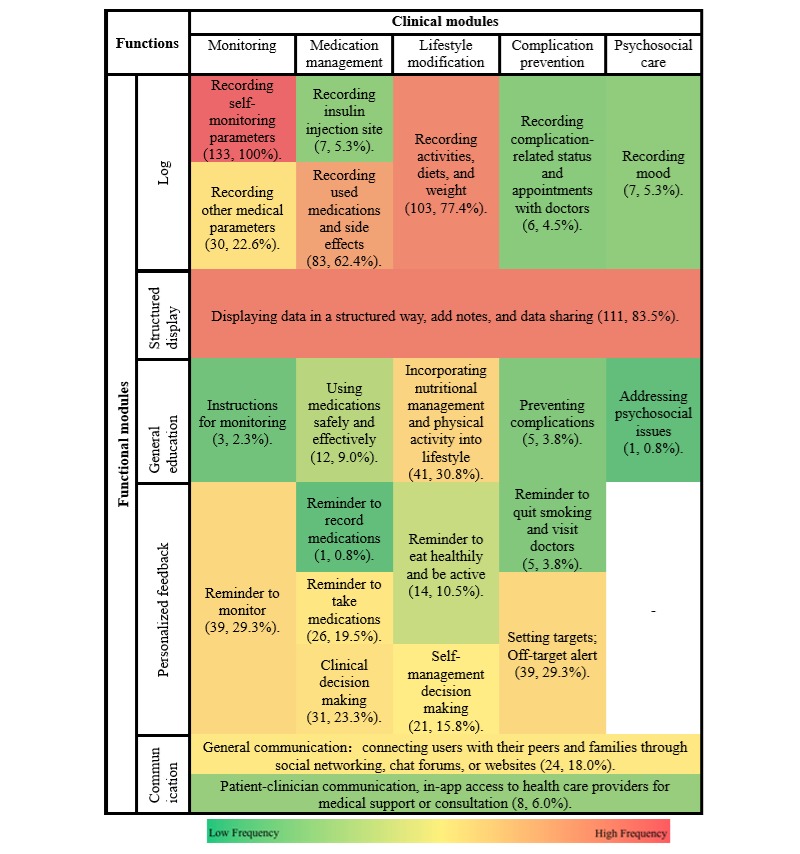
Heatmap of features of 133 US mobile apps for diabetes self-management.

The characteristics of each function provided by the Chinese and US apps have been shown in heatmaps (Figures 2 and 3), and the statistics have been shown in Multimedia Appendix 3.

Chinese and US mobile apps had common functions, including structured display (China: 28/38, 74%; and the United States: 111/133, 83.5%); recording activities, diets, and weights (China: 24/38, 63%; and the United States: 103/133, 77.4%); and recording used medication and side effects (China: 19/38, 50%; and the United States: 83/133, 62.4%).

However, mobile apps between the 2 countries have differences in less common functions. In the 38 Chinese mobile apps, recording complication-related status and appointments with doctors (1/38, less than 3%), preventing complications (3/38, 8%), and setting targets (off-target alert, 3/38, 8%) were the least common functions. In the 133 US mobile apps, addressing psychosocial issues (1/133, 0.8%), reminder to take medications (1/133, 0.8%), and instructions for monitoring (3/133, 2.3%) were the least common functions. Furthermore, none of the 38 Chinese mobile apps included the following 7 functions: recording insulin injections site, recording mood, addressing psychosocial issues, instructions for monitoring, clinical decision making, reminder to record medications, and reminder to quit smoking and visit doctors.

When we took a close look at the distribution of functions in these self-management apps quantitatively, we found that there were differences between apps from China and the United States. Reminder to monitor (China: 5/38, 13% and the United States: 39/133, 29.3%; *P*=.045), setting targets (China: 3/38, 8% and the United States: 39/133, 29.3%; *P*=.007), and clinical decision making (China: 0/38, 0% and the United States: 31/133, 23.3%; *P*=.001) were significantly more common functions in US mobile apps than Chinese apps. However, the following 3 functions were more commonly included in the Chinese apps: patient-clinician communication (China: 26/38, 68% and the United States: 8/133, 6.0%; *P*<.001), using medications safely and effectively (China: 17/38, 45% and the United States: 12/133, 9.0%; *P*<.001), and general communication (China: 15/38, 40% and the United States: 24/133, 18.0%; *P*=.006).

### Risk Assessment of Mobile Apps for Diabetes Between the United States and China

In the United States, 23.3% (31/133) and 65.4% (87/133) of the mobile apps were assessed as high risk and medium risk, respectively. However, none of the Chinese apps was assessed as high risk (Table 2). The difference in the risk of diabetes self-management apps between the United States and China is significant (*P*=.004; Table 2).

**Table 2:** The risk assessment of mobile apps for diabetes self-management between the United States and China.

Risk	United States (N=133), n (%)	China (N=38), n (%)	*P* value
Low	15 (11.3)	3 (8)	.004
Medium	87 (65.4)	35 (92)	.004
High	31 (23.3)	0 (0)	.004

## Discussion

### Principal Findings

This study systematically illustrated the characteristics and functions of in-store mobile apps for diabetes self-management in the United States and China, 2 of the largest app markets, using a predefined taxonomy. It is a useful and reliable tool to categorize functions of these apps despite the country difference [8]. What is more important is that, by using the taxonomy, the study highlights the differences between mobile apps for diabetes self-management in the 2 countries.

### Metadata of Apps

All 133 US apps were categorized as either *medical* or *health and fitness* apps, whereas there are still some Chinese apps (21%, 8/38) that have not been categorized. The reason could be that the FDA risk report and policy recommendations for mobile health technologies suggest that all mobile health–related apps should be divided into 3 categories—general management, health management, and medical devices. Another reason could be that the FDA has the jurisdiction for all mobile health technologies [23]. However, there is no similar regulation in China.

According to a previous survey [19], the needs of Chinese patients with type 1 and type 2 diabetes were significantly different from each other. However, only 1 app in China stated their target users, whereas a few more in the US apps suggested that it was important for app developers to differentiate their products to meet the precise needs of different populations.

### Functions and Modules

Our results show that for diabetes self-management mobile apps, monitoring, lifestyle modification, and medicine management were the 3 most common clinical modules in both China and the United States. It is not surprising that they are the most essential low-risk functions with low technical barriers. However, complication prevention and psychological care were rarely found in the apps from either country although complication prevention was supposed to be associated with more hemoglobin A1_c_ reduction [8,24,25]. The complexity in developing these functions and modules may be the key barriers from being widely adopted.

The functions related to personalized feedback (eg, reminder to monitor, clinical decision making, and setting targets) were more common in US apps than Chinese apps. These functions are based on the built-in algorithms (usually predictive modeling using the collected personal data and probably involving advanced techniques such as artificial intelligence) and provide quick and direct solutions for users’ problems. They are important functions especially when the off-line health care is inaccessible [26]. However, the clinical decision-making model was at high risk [8] and possibly underdeveloped at the moment [27]. The development of personalized feedback modules should be done carefully and with caution, whereas more attempts using different algorithms should be encouraged at the same time.

The communication modules (ie, general communication and patient-clinician communication) are more common in China than in the United States. It is in line with previous population-based survey in China [19], showing that doctor-patient communication is critical to both health providers and patients.

This study adopted our predefined taxonomy [8], which could be different from other systems, for example, Antonio Martinez-Millana et al [26] developed a taxonomy for patients with type 1 diabetes. It comprised 3 hierarchical levels with 10 areas on the first level.

One similar study focusing on Chinese diabetic mobile apps investigated the risk factors related to app use. They suggested that setting recording insulin therapy and dosage in the app might help the patients with type 1 diabetes. They emphasized the importance of the determination of target users before app development [19]. Our study found that 1 Chinese app included recording insulin injection, and only 1 app stated its target type of diabetes. The development of future mobile apps could consider these aspects to improve the effectiveness of mobile apps and user safety.

### Strengths

The strengths of this study are as follows: First, this is the first survey for in-store apps using a predefined taxonomy. Second, the consistency of the predefined taxonomy was strictly tested for its reliability before use. Finally, 2 countries with diverse culture, health care systems, and economic statuses were investigated.

### Limitations

This study also has limitations. First, we only screened the introduction of included in-store apps without downloading and using the apps, which may result in missing out on functional information about the mobile app. However, it is highly consistent with the strategy for a new user to choose an app in the app store. Second, we selected the apps at only one time point, which may miss the longitudinal update of app functions. Further research on monitoring the updates of mobile apps is warranted. Finally, the sample size of apps between China and the United States, accounting for less than 33% (133 vs 38), is unbalanced, which may affect the statistical significance.

### Conclusions

In summary, the in-store mobile apps for diabetes self-management were different between China and the United States. The apps from both countries faced the challenges of lacking evidence-based information, proper risk assessment, and declaration, especially Chinese apps. More Chinese apps included in-app communication modules, whereas more US apps included the clinical decision-making module, which is with high risk. Both complication prevention and psychological care were neglected by the 2 countries. The design of app functions in both countries needs to be optimized, and deep interaction between the app developers and users is recommended. Appropriate surveillance is required to monitor the quality and performance of in-store apps.

## Appendix

Multimedia Appendix 1 The coded taxonomy.

Multimedia Appendix 2 The average of standardized rate of coding error per coder.

Multimedia Appendix 3 The comparison of characteristics of functions provided by mobile apps for self-management of diabetes between the United States and China.

## Abbreviations

FDA: Food and Drug Administration
