# Supplementary material for: A Comparison of Functional Features in Chinese and US Mobile Apps for Diabetes Self-Management: A Systematic Search in App Stores and Content Analysis
Source: JMIR Mhealth Uhealth. 2019 Aug 28;7(8):e13971. doi: 10.2196/13971 (PMC6737884; doi:10.2196/13971)
Supplement: Multimedia Appendix 1 [file mhealth_v7i8e13971_app1.pdf]

Multimedia Appendix 1. The coded taxonomy.

Functional module	Clinical module: (A) Monitoring	(B) Medication management	(C) Lifestyle modification	(D) Complication prevention	(E) Psychosocial care
**(1) Log**	(A1, L) Recording self-monitoring parameters.<br>(A1, M) Recording other medical parameters.	(B1, L) Recording insulin injection site.<br>(B1, M) recording used medications and side effects.	(C1, L) Recording activities, diets, and weight.	(D1, L) Recording complication-related status and appointment with doctors.	(E1, L) Recording mood.
**(2) Structured display**	(2, L) Displaying data in a structured way.				
**(3) General education**	(A3, L) Instructions for monitoring.	(B3, M) Using medications safely and effectively.	(C3, L) Incorporating nutritional management and physical activity into lifestyle.	(D3, M) Preventing complications.	(E3, L) Addressing psychosocial issues.
**(4) Personalized feedback**	(A4, L) Reminder to monitor.	(B4, L) Reminder to recording medications.<br>(B4, M) Reminder to take medications.<br>(B4, H) Clinical decision making.	(C4, L) Reminder to eat healthily and be active.<br>(C4, M) Self-management decision making.	(D4, L) Reminder to quit smoking, visit doctors.<br>(D4, M) Setting targets, off-target alert.	-
**(5) Communication**	(5, L) General communication, connecting users with their peers and families through social networking, chat forums, or websites.<br>(5, M) Patient-clinician communication, in-app access to health care providers support or consultation.				

a. The risk was coded as L, M and H. L- low risk, M-mediate risk, H-high risk.
b. Clinical module was coded as A-E; functional module was coded as 1-5; the crossing functions by clinical module and functional module were coded like A1, A2.
