# Supplementary figures and images for: A Comparison of Functional Features in Chinese and US Mobile Apps for Diabetes Self-Management: A Systematic Search in App Stores and Content Analysis
Source: JMIR Mhealth Uhealth. 2019 Aug 28;7(8):e13971. doi: 10.2196/13971 (PMC6737884; doi:10.2196/13971)

Multimedia Appendix 2. The average of standardized rate of coding error per coder.

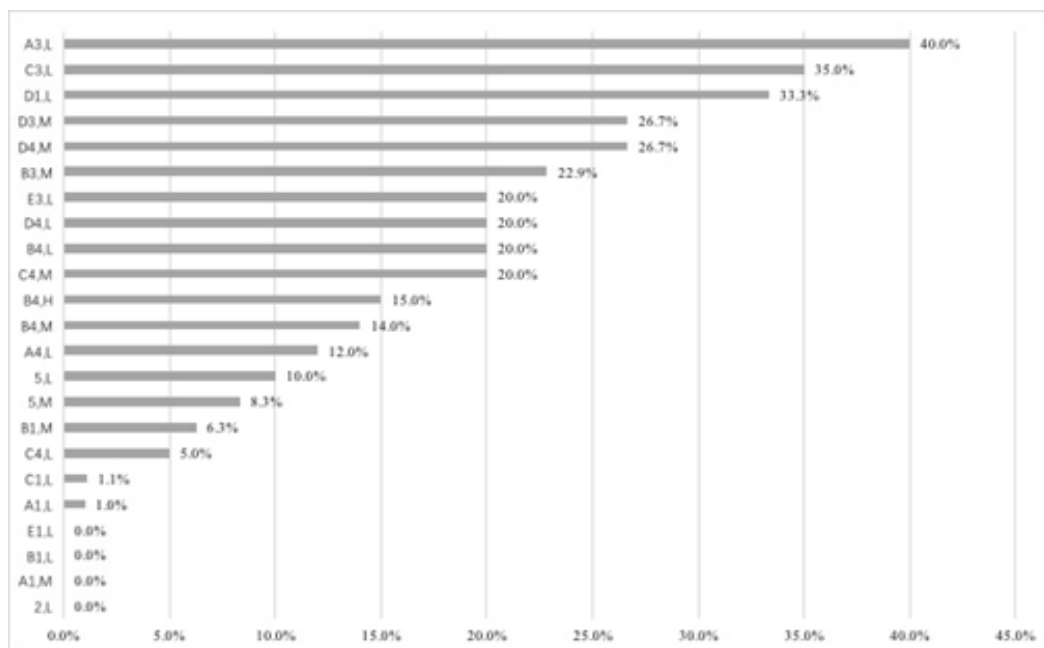

Supplement: Multimedia Appendix 2 [file mhealth_v7i8e13971_app2.pdf]
