# Supplementary material for: A Comparison of Functional Features in Chinese and US Mobile Apps for Diabetes Self-Management: A Systematic Search in App Stores and Content Analysis
Source: JMIR Mhealth Uhealth. 2019 Aug 28;7(8):e13971. doi: 10.2196/13971 (PMC6737884; doi:10.2196/13971)
Supplement: Multimedia Appendix 3 [file mhealth_v7i8e13971_app3.pdf]

Multimedia Appendix 3. The comparison of characteristics of functions provided by mobile apps for diabetes self-management between the United States and China.

| | US (N=133) | China (N=38) | P-value |
|---|---|---|---|
| Recording self-monitoring parameters | 133 (100%) | 38 (100%) | >.99 [a] |
| Recording other medical parameters | 30 (22.6%) | 5 (13%) | .2 [b] |
| Structured display | 111 (83.5%) | 28 (74%) | .17 [b] |
| Instructions for monitoring | 3 (2.3%) | 0 | .6 [a] |
| Recording mood | 7 (5.3%) | 0 | .59 [a] |
| Reminding to monitor | 39 (29.3%) | 5 (13%) | .045 [b] |
| Setting target (Off-target) alerts | 39 (29.3%) | 3 （8%) | .007 [b] |
| Addressing psychosocial issues | 1 (0.8%) | 0 | >.99 [a] |
| General communication | 24 (18%) | 15 (40%) | .006 [b] |
| Patient-clinician communication | 8 (6%) | 26 (68%) | <.001 [b] |
| Recording used medications and side effects | 83 (62.4%) | 19 (50%) | .17 [b] |
| Recording insulin injection site | 7 (5.3%) | 0 | .59 [b] |
| Using medications safely and effectively | 12 (9%) | 17 (45%) | <.001 [b] |
| Reminding to recording medication | 1 (0.8%) | 0 | >.99 [a] |
| Reminding to take medication | 26 (19.5%) | 11 (29%) | .22 [b] |
| Clinical decision making | 31 (23.3%) | 0 | .001 [b] |
| Recording activities, diet and weight | 103 (77.4%) | 24 （63%) | .07 [b] |
| Incorporating nutritional management and physical activity into lifestyle | 41 (10.5%) | 13 （34%) | .69 [b] |
| Reminding to eat healthy and be active | 14 (10.5%) | 4 （11%) | >.99 [a] |
| Self-management decision making | 21 (15.8%) | 6 （16%) | >.99 [b] |
| Recording complications related status and appointments with doctors | 6 (4.5%) | 1 （3%) | >.99 [a] |
| Preventing complications | 5 (3.8%) | 3 （8%) | .5 [a] |
| Reminding to quit smoking, visit doctors | 5 (3.8%) | 0 | .9 [a] |

[a] Fisher exact test.

[b] Chi-square test.
